# Targeting the Conserved Fusion Loop of HAP2 Inhibits the Transmission of *Plasmodium berghei* and *falciparum*

**DOI:** 10.1016/j.celrep.2017.11.024

**Published:** 2017-12-05

**Authors:** Fiona Angrisano, Katarzyna A. Sala, Dari F. Da, Yanjie Liu, Jimin Pei, Nick V. Grishin, William J. Snell, Andrew M. Blagborough

**Affiliations:** 1 Department of Life Sciences, Imperial College of Science, Technology and Medicine, London SW7 2AZ, UK; 2 Institut de Recherche en Sciences de la Santé (IRSS), Bobo Dioulasso, Burkina Faso; 3 Department of Cell Biology and Molecular Genetics, University of Maryland, 4066 Campus Dr., College Park, Maryland 20742, USA; 4 Department of Biophysics and Biochemistry, Howard Hughes Medical Institute, University of Texas Southwestern Medical Center, 6000 Harry Hines Boulevard Dallas, TX 75390-9039, USA

**Keywords:** HAP2, malaria, transmission, fusion, vaccine

## Abstract

Inhibiting transmission of *Plasmodium* is a central strategy in malarial eradication, and the biological process of gamete fusion during fertilization is a proven target for this approach. The lack of a structure or known molecular function of current anti-malarial vaccine targets has previously been a hindrance in the development of transmission-blocking vaccines. Structure/function studies have indicated that the conserved gamete membrane fusion protein HAP2 is a class II viral fusion protein. Here, we demonstrate that targeting a function-critical site of the fusion/*cd* loop with species-specific antibodies reduces *Plasmodium berghei* transmission *in vivo* by 58.9% and *in vitro* fertilization by up to 89.9%. A corresponding reduction in *P. falciparum* transmission (75.5%/36.4% reductions in intensity/prevalence) is observed in complimentary field studies. These results emphasize conserved mechanisms of fusion in *Apicomplexa*, while highlighting an approach to design future anti-malarial transmission-blocking vaccines.

## Highlights

• *Plasmodium* HAP2 is a class II fusion protein
• Class II proteins are targetable to inhibit fertilization/transmission
• A short synthetically generated peptide can induce transmission-blocking immunity

## Graphical Abstract

**Figure:**
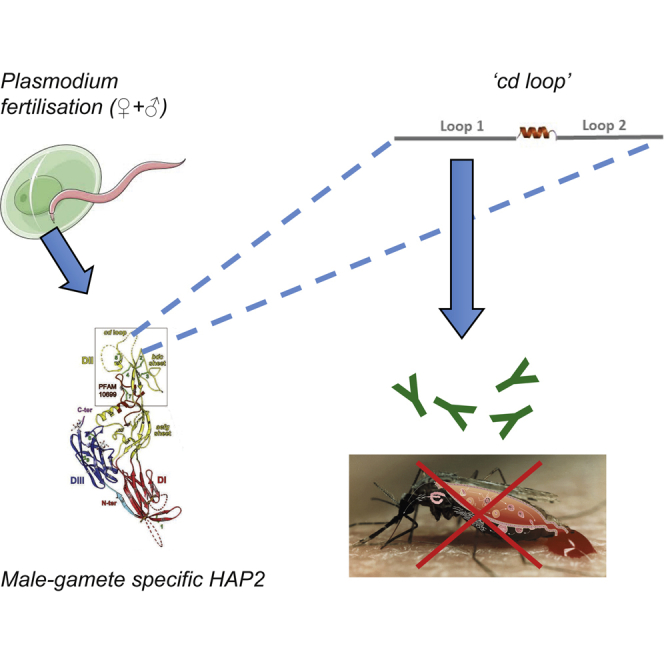

## Teaser

Angrisano et al. find that the HAP2 *cd*-loop can be targeted as an anti-malarial intervention, is immunogenic across multiple plasmodial species, can induce antibodies that specifically recognize the sexual stages of the parasitic life cycle, and can mediate transmission-blocking immunity in the lab and the field.

## Introduction

Malaria remains one of the most prevalent tropical infectious diseases with an estimated 212 million new cases and 429,000 deaths annually (World Health Organization, 2016). *Plasmodium* is transmitted predominantly by mosquitoes of the genus *Anopheles*. Successful transmission of *Plasmodium* from humans to mosquitoes is dependent on the presence of sexually committed (male and female) gametocytes in the peripheral blood, which rapidly undergo the process of activation and differentiate into male (micro) and female (macro) gametes upon uptake by the *Anopheline* vector within a blood meal. Gamete fusion during fertilization is an essential step in the *Plasmodium* life cycle. Fusion is a two-step process, with the first phase encompassing species-specific recognition of male and female gametes via surface-localized membrane proteins. In *Plasmodium*, only three proteins have been discovered that have a demonstrable role in the mutual recognition of gametes; P48/45, P47, and P230 (van Dijk et al., 2001, van Dijk et al., 2010, van Schaijk et al., 2006). This initial recognition/adhesion step is postulated to initiate a signal transduction cascade that activates the microgamete and exposes new, fusogenic regions of the sperm plasma membrane (Liu et al., 2008). Adhesion is rapidly followed by the second phase of fertilization; merger of lipid bilayers to achieve cytoplasmic continuity. The conserved male-specific protein, HAP2/GCS1 is essential for mediating merger of bilayers (Mori et al., 2006, von Besser et al., 2006, Liu et al., 2008). Following fertilization, the resulting zygotes develop into motile ookinetes, establishing infection in the insect host. Despite the obvious biological importance of fertilization and its proven previous targeting as a potential point to disrupt the parasitic life cycle with therapeutics (Carter, 2001, van Dijk et al., 2010, Blagborough et al., 2013a), our knowledge of the cellular and molecular mechanisms underlying gamete fusion in *Plasmodium* is surprisingly sparse.

**Figure 1:**
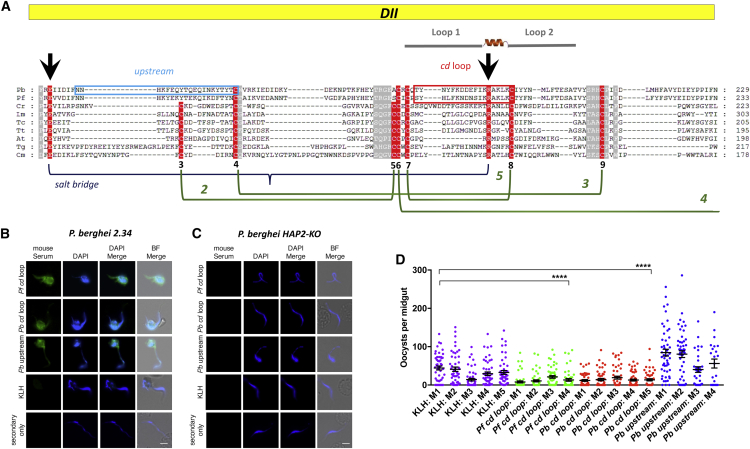
Identification of a Putative *Plasmodium* HAP2 Fusion Loop and Assessment of *In Vivo* Transmission-Blocking Efficacy by Direct Feeding Assays in Immunized Mice (A) Domain II (DII) schematic (yellow box) above alignment of the cysteine-rich region of HAP2 proteins from *P. berghei* (*Pb*), *P. falciparum* (*Pf*), *Chlamydomonas reinhardtii* (*Cr*), *Leishmania major* (*Lm*), *Tribolum castaneum* (*Tc*), *Tetrahymena thermophile* (*Tt*), *Arabidopsis thaliana* (*At*), *Toxoplasma gondii* (*Tg*), and *Crytosporidium muris* (*Cm*). Conserved and semi-conserved residues are in white font on a red or silver background, respectively. Conserved cysteine residues are numbered sequentially below the alignment, and their disulfide connectivities are drawn in green with disulfide bonds numbered (see Fédry et al., 2017). The conserved salt bridge between R and E (arrowheads above the sequence) is drawn in blue below the alignment. Loops 1 and 2 of the cd loop are highlighted in gray and separated by an alpha helix. A region upstream of the predicted fusion loop to which peptides and antibodies were generated is highlighted in blue and is referred to as *Pb* upstream. Highlighted in red is the short (18 aa) region within the predicted *P. berghei* and *P. falciparum* bipartite fusion loops to which peptides and antibodies were generated and is referred to as the *Pb cd* loop and *Pf cd* loop, respectively. (B and C) IFA with serum from mice immunized with either *Pf cd* loop (aas 178–194), *Pb cd* loop (aas 174–191), *Pb* upstream (aas 123–142), or KLH (negative control) recognizes WT *P. berghei* ANKA male gametocytes/gametes (B). Staining is absent in KLH control serum and in all IFAs performed with *P. berghei* HAP2-KO gametocytes/gametes (C). Scale bar, 5 μm. (D) Three cohorts of five mice were immunized with KLH, *Pf cd* loop, *Pb cd* loop, or *Pb* upstream (NB 1 mouse from *Pf cd* loop and *Pb* upstream were culled on veterinary advice before end of experiment). Each cohort was challenged with WT *P. berghei* 2.34, and determination of transmission blockade was performed by DFA. Individual data points represent the number of oocysts found in individual mosquitoes 12 days post-feeding. Horizontal bars indicate mean intensity of infection, while error bars indicate SEM within individual samples. Asterisks indicate p value < 0.05 Mann-Whitney U test.

The membrane fusogen HAP2 was originally identified in a screen for male sterility in the flowering plant *Arabidopsis thaliana* (von Besser et al., 2006) and later—also under the name GCS1 in *Lilium longflorum* pollen (Mori et al., 2006)—as a sperm-specific protein shown to be required at an unidentified step in sperm-egg fusion. It is highly conserved and observed in a wide range of species, including pathogenic and non-pathogenic protists, choanoflagellates, algae, higher plants, and metazoans (Liu et al., 2008, Ning et al., 2013). In *Plasmodium*, targeted disruption of HAP2/GCS1 in *P. berghei* affects the male gametes ability to fuse with female gametes and is required for successful fertilization of the sexual stages of the parasite (Blagborough and Sinden, 2009). To date, there has been limited functional or structural evidence to examine the role of HAP2. Recent studies on HAP2 of the green alga *Chlamydomonas* determined the atomic structure of HAP2, demonstrating that it is a eukaryotic class II fusion protein, with homology to somatic and viral fusogens. Class II fusion proteins are present in a wide range of eukaryotic/viral species of veterinary and clinical importance (e.g., dengue, yellow fever, West Nile viruses, alphaviruses, Eimeria, and Zika) (Fédry et al., 2017, Pinello et al., 2017, Valansi et al., 2017). Using a conserved mechanism, the core function of class II fusion proteins is to mediate exoplasmic membrane fusion and merger of lipid bilayers either unilaterally or bilaterally (Podbilewicz et al., 2006, Podbilewicz, 2014). In viral systems, conformational changes triggered during virus-host interactions ultimately lead to re-configuration of proteins into trimers, with the hydrophobic “fusion loop” (*cd* loop) inserted into the target cell membrane. Subsequent conformational changes, termed hairpining, bring the two membrane anchors and their associated bilayers together, followed by complex biophysical rearrangements of the lipid bilayers to consummate bilayer fusion. Previously, concerted bioinformatic, functional, and X-ray structural analyses of HAP2 from *Chlamydomonas reinhardtii* were performed (Fédry et al., 2017). Specifically, HHpred protein homology detection methods identified a cysteine-rich polypeptide segment in *C. reinhardtii* HAP2 ectodomain (amino acids [aas] 170–204) that exhibited alignment to the fusion loop region of the flavivirus envelope protein E. Further analysis of HAP2 orthologs showed that the sequence in this region is variable (Figure 1), with a number of deletions and insertions, and is framed at each side by relatively conserved segments: (*C. reinhardtii* residues 159–167 and residues 208–219) (Figure 1). Only two aas, R185 and C190, within the HHPred identified *Chlamydomonas* “cd loop” segment are conserved across all species, suggesting that they may play a potential role in HAP2 function. Subsequent mutational studies (Fédry et al., 2017) demonstrated this short section was essential for HAP2 function in *C. reinhardtii*. This region (aas 170–204) corresponds to aas 174–205 (*P. berghei*) and aas 178–207 (*P. falciparum*). Related studies using homology modeling and mutational analysis with *Tetrahymena thermophila* (Pinello et al., 2017) and *Arabidopsis thaliana* (Valansi et al., 2017) also indicate that HAP2 is a class II fusion protein, and the *cd* loop is essential for fusion. The structure of *Chlamydomonas* HAP2 suggests that the *cd* loop is bipartite, with the two parts split by a small alpha helix (Fédry et al., 2017). Given this specific structure/function biological activity observed during the study of HAP2 in *Tetrahymena*, *Arabidopsis*, and *Chlamydomonas*, and the general conservation of the protein class across eukaryotes, higher plants, and viruses, it is postulated that these findings could be successfully extrapolated to multiple pathogens to design interventions to block fertilization. In the case of *Plasmodium*, this would logically result in a blockade in malarial transmission.

It is widely accepted that to achieve malarial eradication, it will be necessary to use interventions that inhibit the transmission of parasites from humans to mosquitoes (malERA Consultative Group on Health Systems and Operational Research, 2011). A potential manner of achieving this is by targeting *Plasmodium* using transmission-blocking vaccines (TBVs) against parasitic sexual stages (Sauerwein and Bousema, 2015). The comparatively short lifespan, increased fragility, and availability of proven surface-localized antigens on the male gamete of *Plasmodium* make targeting this stage of the life cycle a potential method of inhibiting transmission (Vermeulen et al., 1985). Based on first principles, an optimal TBV target protein would be localized at the parasitic cell surface, conserved across all five malaria species that infect humans, and the function and structure of such a target would be known, thereby allowing rational selection of immunologically vulnerable domains. Antibodies targeted to multiple TBV targets have confirmed localization to proteins located on the surface of the plasma membrane of the male gamete (P230, P48/45) (van Dijk et al., 2001, van Dijk et al., 2010, van Schaijk et al., 2006), clearly indicating the value of targeting this stage of the parasite life cycle. HAP2 also fulfils these criteria. Experiments demonstrating that use of recombinant HAP2 protein encompassing aas 355–609, which lack the *cd* loop, can induce anti-*Plasmodium* HAP2 transmission-blocking antibodies in mice have been reported previously (Blagborough and Sinden, 2009, Miura et al., 2013). However, the expression systems used were not sufficient for viable clinical development, and the mechanism of induced blockade was not examined.

To investigate the specific role of *Plasmodium* HAP2 fusion loop in gamete fertilization and its ability to mediate a transmission-blocking effect, we generated keyhole limpet hemocyanin (KLH)-conjugated (Helling et al., 1995, Ragupathi et al., 2002) *cd* loop peptides, and subsequently, antibodies to both *P. berghei* and *P. falciparum cd* loops, as well as control “non-*cd* loop” peptides/antibodies specific to a region upstream of the *P. berghei* fusion loop (Figure 1). These regions were selected because: (1) they contained the only conserved residues (corresponding to *C. reinhardtii* R185 and C190) within the identified fusion loop (Fédry et al., 2017), and thus, are potentially required for membrane fusion; (2) this region additionally covered the first strand only of the bipartite fusion loop, allowing us to examine its potential function more closely; and (3) it was possible to stably synthesize the respective peptide for immunization. Conversely, the “non-*cd* loop” peptide region (aas 122–142) (Figure 1) was selected as an appropriate negative control for the anti-*cd* loop domain because: (1) it lies within domain II of HAP2, but outside of the predicted *cd* loop, (2) it contains a single conserved cysteine (as does the “cd loop peptide”); and (3) it does not contain the two key resides (R168/E117 in *P. berghei*) postulated to be responsible for salt bridge formation in the plasmodial HAP2 ectodomain, and thus, organization of the target membrane-interacting region.

Using these tools, we show that immunizations with peptides targeting this fusion loop in *P. falciparum* and *P. berghei* elicits effective specific humoral immune responses. The resulting sera recognize the surface of exflagellating male gametes with native conformation and confer significant transmission-blocking activity when assessed *in vivo* by allowing direct feeding of mosquitoes on immunized mice following parasite challenge. We further demonstrate that anti-*cd* loop antibodies inhibit parasite fertilization and confer significant transmission-blocking activity *in vitro*, by assessing successful fertilization events in culture, and *ex vivo*, by adding antibody to parasite-infected blood on which mosquitoes are allowed to feed. The latter method, termed the standard membrane feeding assay (SMFA), is the current primary assay to assess TBV efficacy (Churcher et al., 2012). Finally, utilizing the direct membrane feeding assay (DMFA) on blood samples from infected African blood donors (Sauerwein and Bousema, 2015), we demonstrate that anti-*P. falciparum* HAP2 *cd* loop antibodies result in potent reduction in malaria transmission in the field over a range of naturally occurring infection densities. This data comprise a primary example of linking potential protein structure/function to efficacy, in the context of a transmission-blocking vaccine. Functional and structural information regarding the commonly quoted current TBV targets (P25, P48/45, P230) is still sparse and is largely un-linked to antibody efficacy. Our results demonstrate that the specific mechanism of class II fusion is targetable to inhibit the progression of the *Plasmodium* life cycle, specifically at the point of fertilization/transmission. We use a short, synthetically generated peptide to induce transmission-blocking immunity in lab and field studies. The use of a small, cheap, synthetic peptide with optimization and further work could circumvent many problems related to traditional heterologous protein expression when considering vaccine targets. These results, generated using a conjugated peptide-based vaccination, provide evidence that *Plasmodium* HAP2 may act as a class II fusogen and documents the conserved mechanisms of membrane fusion within *Plasmodium* and across multiple kingdoms. These results emphasize the potential for of the use of rationally selected immunogens to enable the development of anti-malarial transmission-blocking vaccines in the future.

## Results

### Analysis of HAP2 Primary Sequence in *Plasmodium*

Recent bioinformatic and structural analysis of *C. reinhardtii* HAP2 identified similarities to eukaryotic/viral class II fusion proteins, revealing a specific segment 42-residue region within a cysteine-rich portion of HAP2 that corresponded to the predicted fusion loop (Fédry et al., 2017). An alignment of a section of the HAP2 domain II (DII) from *P. berghei* (*Pb*), *P. falciparum* (*Pf*), *Chlamydomonas reinhardtii* (*Cr*), *Leishmania major* (*Lm*), *Tribolum castaneum* (*Tc*), *Tetrahymena thermophile* (*Tt*), *Arabidopsis thaliana* (*At*), *Toxoplasma gondii* (*Tg*), and *Crytosporidium muris* (*Cm*). Figure 1A reveals that the sequence in this region is variable over multiple taxa, with multiple deletions, substitutions, and insertions observed throughout the alignment, while predicted disulfide bonds and salt bridge as described in Fédry et al. (2017) are largely conserved. The bipartite *cd* loop region (predicted by homology to *Chlamydomonas* HAP2) is indicated on Figure 1. An 18-residue region (highlighted in red—predicted by homology to *Chlamydomonas* HAP2) (Fédry et al., 2017) within the predicted *P. berghei* and *P. falciparum* fusion loop was used to generate peptides and raise antibodies to examine the ability of the *cd* loop to mediate an anti-parasitic transmission-blocking response. Comparison of this region between *Chlamydomonas* and *P. berghei* HAP2 demonstrates a total 22% residue conservation and 36% sequence similarity at the primary sequence level. Correspondingly, KLH-conjugated peptides for immunization, and specific anti-*cd* loop antibodies were generated against this region for both the *P. berghei* and *P. falciparum* sequence (referred to as the *Pb cd* loop and *Pf cd* loop, respectively). Additionally, to control for non-specific anti-*cd* loop effects, KLH-conjugated peptide and antibody against a 20-residue region (highlighted in blue) in domain II of the protein was generated. This region is located within domain II located 31 aa upstream of the *cd* loop and serves as an internal control. It contains a conserved cysteine but does not include either the conserved R or E involved in forming the salt bridge.

### Immunization with *P. falciparum* and *P. berghei* HAP2 Peptides Induces HAP2-Specific Antibodies

**Figure 2:**
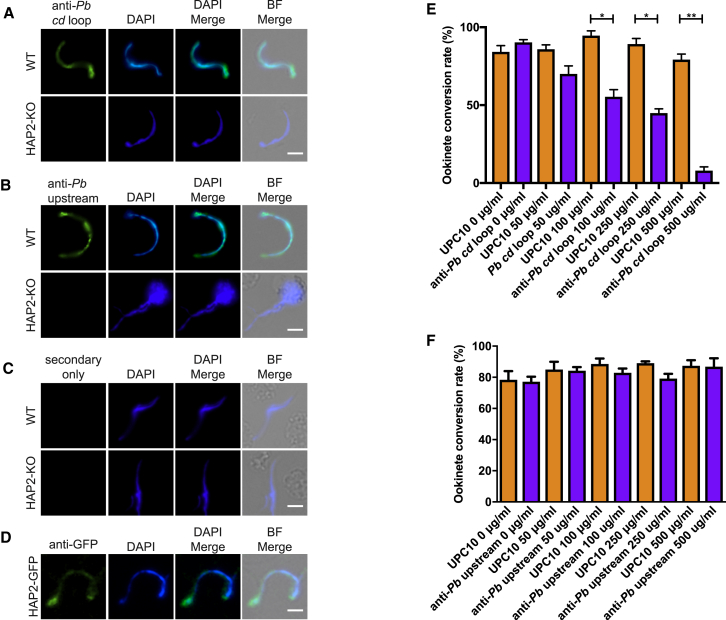
Evaluation of *Pb cd* Loop and *Pb* Upstream Antibodies to Inhibit Ookinete Conversion (A and B) IFA of WT *P. berghei* ANKA and *P. berghei* HAP2-KO male gametes with (A) anti-*Pb cd* loop and (B) anti-*Pb* upstream. (C) Secondary-only control antibodies (green) DAPI (blue). (D) IFA of HAP2-GFP male gametes with anti-GFP shows a similar staining pattern to that obtained using anti-*Pb cd* loop and anti-upstream antibodies. Scale bars, 5 μm. (E and F) *In vitro* ookinete development assays with anti-*Pb cd* loop (E) and anti-upstream (F) antibodies compared to negative control antibody UPC10 at concentrations of 0, 50, 100, 250, and 500 μg/mL. ^∗^p value < 0.05 paired t test. Error bars indicated SEM within individual samples.

Peptide regions of HAP2 that correspond to the predicted fusion loop in *P. falciparum* (*Pf cd* loop) and *P. berghei* (*Pb cd* loop), along with a peptide upstream of the predicted fusion loop in *P. berghei* (*Pb upstream*) were generated and chemically coupled to a KLH carrier protein (Yenzym, USA). Conjugation to KLH was carried out in order to stabilize the peptide and elicit a stronger and more persistent immunological response. To examine the potential induction of HAP2-specific serum to native protein on the surface of male gametes following immunization with *Pf cd loop*, *Pb cd loop*, *Pb upstream*, and KLH (negative control) immunogens, we performed an immunofluorescence assay (IFA) on non-permeabilized wild-type (WT) *P. berghei* 2.34, using pooled serum generated from immunized mice. Surface staining was observed on activated, exflagellating male gametes and free-floating male gametes in *Pf cd* loop, *Pb cd* loop, and *Pb upstream* immunization groups (Figure 1B), confirming the ability of the resulting sera (raised against both *P. falciparum*- and *P. berghei*-derived peptides) to recognize native HAP2 on the surface of male gametes. Staining was not observed in the control carrier protein KLH immunization group, indicating KLH does not induce a detectable anti-parasitic response following immunization. Staining was also not observed when this serum was used in an IFA with HAP2-KO line (Figure 2C). The ability of serum generated following immunization against the *Pf cd loop* immunized group to recognize the surface of the *P. berghei* gamete highlights the general conservation of the *cd* loop in multiple *Plasmodium* species.

### *In Vivo* Immunization against *P. falciparum* and *P. berghei cd* Loop Blocks Malarial Transmission

**Figure 3:**
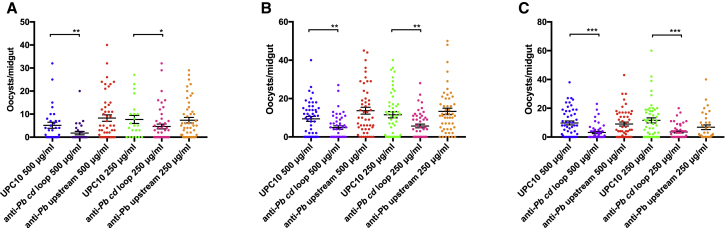
Transmission-Blocking Efficacy of *Pb cd* Loop and *Pb* Upstream Antibodies in SMFA (A–C) Triplicate SMFAs with anti-*Pb cd* loop and anti-*Pb* upstream antibodies compared with negative control antibody UPC10 at concentrations of 250 and 500 μg/mL replicate 1 (A), replicate 2 (B), and replicate 3 (C). Individual data points represent the number of oocysts found in individual mosquitoes 12 days post-feeding. Horizontal bars indicate mean intensity of infection, while error bars indicate SEM within individual samples. ^∗^p value < 0.05 Mann-Whitney U test. ns, p value not significant.

**Figure 4:**
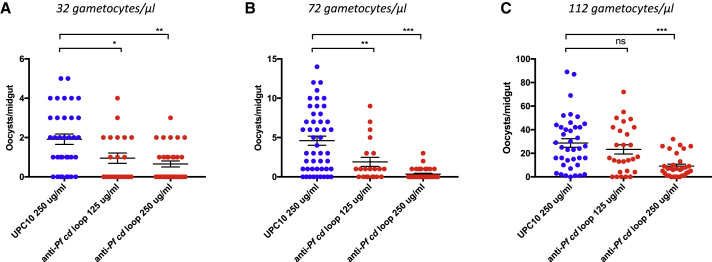
Assessment of Transmission-Blocking Efficacy of *Pf cd* Loop Antibodies in Gametocyte-Positive Patient Blood from Burkina Faso Using DMFA (A–C) DMFA at three naturally occurring gametocyte densities; 32 gametocytes/μl (A), 72 gametocytes/μl (B), and 112 gametocytes/μl (C) with anti-*Pf cd* loop antibody at concentrations of 125 and 250 μg/mL compared with negative control antibody UPC10 at 250 μg/mL. Individual data points represent the number of oocysts found in individual mosquitoes 7 days post-feeding. Horizontal bars indicate mean intensity of infection, while error bars indicate SEM within individual samples. ^∗^p value < 0.05 Mann-Whitney U test. ns, not significant.

To examine the effect of immunization with conjugated anti *cd* loop peptide on transmission *in vivo*, groups of immunized mice (n = 5) were infected with *P. berghei* ANKA 2.34, following which, *An. stephensi* mosquitoes were allowed to feed directly on immunized and challenged hosts (Figure 1D; Table 1). Mosquitoes that fed on KLH-immunized mice displayed an average oocyst intensity of 32.39, whereas following subcutaneous immunization with either KLH-conjugated *Pf cd* loop peptide or KLH-conjugated *Pb cd* loop peptide the mean intensity was reduced to 13.31 and 14.34 oocysts/midgut, respectively. Significant overall mean reductions of 58.90% and 55.71% were observed, respectively (p ≤ 0.0001). Correspondingly, a mean infection prevalence of 80% in the KLH control cohort was reduced to 49% in the *Pf cd* loop and 53% in the *Pb cd* loop immunized cohort, resulting in a mean reduction of 38.44% and 33.16%, respectively (p ≤ 0.0001). No statistical significance was observed between control (WT non-immunized) cohort and KLH immunized mice. Significantly, the group immunized with KLH-conjugated *Pb* upstream peptide exhibited no transmission-blocking activity, in terms of either infection intensity or prevalence. The *Pb* upstream cohort suggests potential enhancement of transmission. Discussion is currently active within the field to elucidate if antibody-mediated enhancement is a real phenomenon, or an artifact of current assays. This must be investigated robustly with larger cohorts. Enhancement was not demonstrated with other transmission-blocking assays within this study (Figures 3 and 4). Despite the relatively small regions of the HAP2 ectodomain targeted with these peptide immunizations, we see a significant transmission-blocking effect with peptides targeted to the *cd* fusion loop, compared to peptides targeted to an upstream region in domain II of HAP2. This demonstrates that the *cd* loop of *P. berghei*, predicted to be critical for gamete fusion in multiple divergent species, is a potential anti-parasitic transmission-blocking vaccine target.

**Table 1:** Mean *In Vivo* Evaluation of Transmission-Blocking Effect of *Pf cd* Loop, *Pb cd* Loop, and *Pb* Upstream

	Control	KLH	*Pf cd* Loop	*Pb cd* Loop	*Pb* Upstream
Mean intensity	39.38	32.39	13.31	14.34	65.31
Mean prevalence	96	80	49	53	87
Inhibition in intensity (%)		17.76	58.90^a^	55.71^a^	−101.65^a^
Inhibition in prevalence (%)		16.99	38.44^b^	33.17^b^	−9.74

Inhibition in intensity (mean number of oocysts per midgut) and prevalence in groups of mice immunized with each affinity-purified antibody were calculated with respect to the appropriate, grouped control mice.

a p < 0.05, Mann-Whitney U test.

b p < 0.05, Fisher’s exact test.

### Antibodies Targeting the *P. berghei cd* Loop Inhibit Fertilization *In Vitro*

To further examine the ability of the *cd* loop to initiate a specific anti-parasitic transmission-blocking response, affinity-purified antibodies, raised in rabbits against both the *Pb cd* loop (anti-*Pb cd* loop) and *Pb* upstream (anti-*Pb* upstream) regions were examined *in vitro*. Anti-*Pb cd* loop and anti-*Pb* upstream antibodies both recognized the surface of exflagellating and free-floating *P. berghei* male gametes by IFA. Staining was absent in assays with HAP2-KO parasite lines, indicating the ability of these antibodies to recognize natively folded HAP2 on the gamete surface (Figures 2A and 2B). Control antibody/secondary only controls resulted in no observable staining (Figure 2C). The observed staining along the length of the gamete is similar to that of a previously described and characterized HAP2-GFP line (Figure 2D). To confirm the ability of antibodies targeted against these domains to act as transmission-blocking agents, we performed *in vitro* ookinete conversion assays. Antibodies raised against the anti-*Pb cd* loop inhibited ookinete conversion in a dose-dependent manner. At concentrations of 100, 250, and 500 μg/mL, antibody supplemented cultures exhibited conversion rates of 55.34%, 44.87%, and 7.94%, respectively. This equates to an inhibition in ookinete formation of 41.50% at 100 μg/mL, 49.71% at 250 μg/mL, and 89.97% at 500 μg/mL. In contrast, as expected (and previously demonstrated) (Blagborough et al., 2013b), the presence of UPC10 (negative control) had no effect on ookinete conversion rate (100 μg/mL, 94.61%; 250 μg/mL, 89.22%; 500 μg/mL, 79.22%) (Figure 2E). Antibodies raised against the upstream region (i.e., anti-*Pb* upstream) had no discernible effect on ookinete conversion rate (100 μg/mL, 82.88%; 250 μg/mL, 79.11%; 500 μg/mL,; 86.77%) at any dilution examined (Figure 2F). Unlike the results from the direct feeding assay (DFA), enhancement is not observed, potentially due to differences in sensitivity or the respective biology efficacy of *in vitro* and *in vivo* assays.

### Antibodies Targeting *P. berghei cd* Loop Inhibit Transmission *Ex Vivo*

The transmission-blocking activity of anti-Pb cd loop and anti-Pb upstream antibodies were assessed in triplicate by SMFA (Figures 3A–3C). Given the *in vitro* results observed previously (Figure 2E), we assessed the *in vivo* transmission-blocking ability of these antibodies at the two highest concentrations (250 and 500 μg/mL) where an effect in the *in vitro* ookinete assay was demonstrated. Anti-Pb cd loop antibodies inhibited P. berghei transmission at both concentrations tested in a dose-dependent manner. At a concentration of 250 μg/mL, anti-Pb cd loop antibodies inhibited oocyst intensity by a mean of 53.32%, p ≤ 0.05 and reduced prevalence of infection by 27.26%, p ≤ 0.05. At 500 μg/mL, a 60.57%/38.97% reduction in terms of both intensity/prevalence was observed, p ≤ 0.05 (Table 2). Consistent with previous results, anti-Pb upstream antibodies did not inhibit transmission.

**Table 2:** Mean *Ex Vivo* Evaluation of Transmission-Blocking Effect of *Pb cd* Loop and *Pb* Upstream

	Anti-*Pb* cd Loop (500 μg/ml)	Anti-*Pb* cd Loop (250 μg/mL)	Anti-*Pb* Upstream (500 μg/mL)	Anti-*Pb* Upstream (250 μg/mL)
Inhibition in intensity (%)	60.57^a^	53.32^a^	−32.91	14.33
Inhibition in prevalence (%)	38.97^b^	27.26^b^	−9.66	−1.10

The mean (from three replicates) inhibition in intensity (mean number of oocysts per midgut) and prevalence with concentrations of anti-*Pb cd* loop and anti-*Pb* upstream at 250 and 500 μg/mL were calculated with respect to appropriate negative control at the relevant concentration.

a p < 0.05, Mann-Whitney U test.

b p < 0.05, Fisher’s exact test.

### Antibodies Targeting *P. falciparum cd* Loop Inhibit Transmission in the Field across a Range of Naturally Occurring Gametocyte Densities

To assess the ability of *cd* loop antibodies to block transmission of malarial field isolates, *P. falciparum* gametocytes were collected from naturally infected volunteers recruited in malaria endemic localities, and DMFA subsequently performed with purified *Pf cd* loop antibodies at two concentrations (Figures 4A–4C). Each patient in the field had a unique gameocyte density. Multiple naturally occurring gametocyte densities (n = 3) were assessed to examine a range of challenge contexts. In triplicate experiments, significant dose-dependent transmission-blocking efficacy was observed at both 125 and 250 μg/mL, with maximal mean reductions in intensity/prevalence of 75.50%/36.46% observed at 250 μg/mL (p ≤ 0.0001) (Table 3).

**Table 3:** Mean *Ex Vivo* Evaluation of Transmission-Blocking Effect of *Pf cd* Loop Antibodies

	Anti-*Pf cd Loop* (125 μg/mL)	Anti-*Pf cd Loop* (250 μg/mL)
Inhibition in intensity (%)	42.63^a^	75.50^a^
Inhibition in prevalence (%)	24.09^b^	36.46^b^

The mean (from three replicates) changes in intensity (mean number of oocysts per midgut) and prevalence with anti-*Pf cd* loop antibody at 125 and 250 μg/mL were calculated with respect to appropriate negative control antibody UPC10 at the highest concentration tested.

a p < 0.05, Mann-Whitney U test.

b p < 0.05, Fisher’s exact test.

## Discussion

The conserved transmembrane protein HAP2 is essential for gamete fusion during fertilization in multiple organisms across kingdoms, specifically during bilayer merger. More broadly, class II fusion proteins have been demonstrated as key initiators of membrane fusion in a range of eukaryotic and viral taxa. This class of protein is of increasing clinical interest, as activity is essential for successful viral entry of multiple pathogens into target cells by initiating merger of lipid bilayers (Kielian, 2006, Kielian and Rey, 2006, Modis, 2013). Previous studies have shown that HAP2 is restricted to expression in male gametes in sexually dimorphic species (Liu et al., 2008), and expression appears to be localized to regions of the plasma membrane where fusion occurs (Liu et al., 2008). Genetic ablation of *HAP2* or gross mutagenesis of the HAP2 ectodomain in a range of species across multiple taxa abolishes fertilization but not gamete binding (Liu et al., 2008, Blagborough and Sinden, 2009), leading to the conclusion that HAP2 is a conserved male-specific fertility protein, responsible for mediating cell-cell fusion and resulting cytoplasmic continuity. However, until recently, evidence regarding a specific cellular role, structure, or mechanism of action to HAP2 in any species was lacking. Recent studies in *Chlamydomonas*, *Tetrahymena*, and *Arabidopsis* have positively identified HAP2 as a class II fusion protein (Fédry et al., 2017, Pinello et al., 2017, Valansi et al., 2017). Each HAP2 has three distinct domains, with domains II and III implicated in class II membrane fusion. A conserved, 46-residue hydrophobic-rich region within domain II was positively identified as the fusion loop using multiple methods across multiple organisms (Fédry et al., 2017, Pinello et al., 2017, Valansi et al., 2017). Following a (currently uncharacterized) trigger, it is hypothesized that the fusion loop becomes exposed on the membrane surface, leading to the alignment of protein subunits parallel to each other, favoring trimerization. Polar residues on the fusion loop are subsequently inserted into the target membrane, followed by a conformational change in HAP2 domain III that distorts the target membrane, leading to hemifusion and then fusion/cytoplasmic continuity.

HAP2 was originally identified and characterized specifically in *Plasmodium* via comparative studies in *Chlamydomonas* (Liu et al., 2008, Blagborough and Sinden, 2009), which demonstrated its exclusive expression in the male gamete and its necessity for transmission to the mosquito host. Following studies examined its role as a potential TBV target, with a strategy that used recombinant *Plasmodium* HAP2 fragments to induce transmission-blocking immunity. In both of these studies, fractions of the protein were successfully expressed in heterologous systems and demonstrated significant transmission-blocking efficacy in *P. berghei* and *P. falciparum*. However, expression systems used, yield and purity were not appropriate for clinical development. The mechanism of induced blockade was not examined in these initial experiments. Given the recent identification of HAP2 as a class II fusion protein and the identification of a fusion/*cd* loop region in the protein sequence, we describe here the use of a KLH-conjugated peptide, spanning the postulated plasmodial HAP2 *cd* loop, as an antigen. We demonstrate that immunization of rodents with this simple immunogen, synthesized without any requirement for (traditionally problematic in *Plasmodium*) (malERA Consultative Group on Health Systems and Operational Research, 2011) heterologous protein expression, results in the induction of antibodies that recognize the target immunogen in its native conformation on the surface of the male gamete and also in the induction of significant transmission blockade *in vivo*. We further show that affinity-purified polyclonal antibodies, raised in rabbits against KLH-conjugated *P. berghei cd* loop peptides, result in potent, dose-responsive 89.97% inhibition of ookinete formation in culture and in significant, dose-responsive *ex vivo* inhibition of transmission in the SMFA. Furthermore, we show that anti-*P. falciparum* fusion loop antibodies are capable of inducing significant (up to 75.5% reduction in intensity) transmission-blocking efficacy, assessed on field isolates of *P. falciparum* (human malaria) from infected blood donors at a range of naturally occurring gametocyte densities, in the DMFA. Although efficacy at this level does not result in total interruption of transmission from human to mosquito, previous studies using population transmission-based animal models has demonstrated that interventions with this efficacy could profoundly contribute to elimination campaigns, especially at lower transmission intensities (Blagborough and Sinden, 2009).

These results provide evidence that the *Plasmodium cd* loop is broadly immunogenic across two plasmodial species, can induce antibodies that specifically recognize the sexual stages of the parasitic life cycle and can mediate transmission-blocking immunity in the lab and the field. These data also provide supporting evidence to add to the multiple extensive studies indicating that HAP2 is a class II fusion protein with an exposed fusion/*cd* loop, and targeting the *cd* loop is a potential viable therapeutic target across a range of taxa. Previous experiments on *Chlamydomonas* have demonstrated that pre-incubation of *minus* gametes with an anti-HAP2_168–190_ (i.e., anti-*cd* loop) polyclonal antibody had no effect on motility or adhesion, but inhibited gamete fusion by 75% (Fédry et al., 2017). Additionally, a peptide derived from the homologous region of *Tetrahymena thermophila* HAP2 demonstrated properties characteristic of a fusion loop (Pinello et al., 2017). Human antibodies against the class II glycoprotein E fusion protein in dengue virus (serotype 2) also demonstrate broadly neutralizing activity by binding to the exposed main chain of the fusion loop and the conserved glycan chain (Rouvinski et al., 2015). Antibodies against this dengue epitope also mediate cross-species neutralization against Zika virus by targeting the site of the envelope protein dimer with the precursor membrane protein during virus maturation (Barba-Spaeth et al., 2016). Our data, obtained in two species of *Plasmodium*, are compatible with these findings and the crystal structure and mutagenesis data previously outlined in extensive study of class II fusion proteins and HAP2 in multiple species (Fédry et al., 2017, Pinello et al., 2017, Valansi et al., 2017). Specifically, our results suggest that the conserved region within the hypothesized *Plasmodium cd* loop peptide plays a key role during membrane fusion, and in combination with the data presented in Fédry et al. (2017), strongly suggest that R185 (R168 in *P. berghei*) is vital for gamete fusion. Specifically, this conserved arginine is postulated to organize the target membrane-interacting region of HAP2 by establishing a salt bridge with a conserved upstream glutamic acid (E117 in *P. berghei*) (Figure 1). Here, we demonstrate that antibody-mediated targeting of this region specifically inhibits malarial transmission, whereas interference with other regions containing putative conserved cystines within domain II (e.g., the “upstream” region) (Figure 1) (Fédry et al., 2017) does not result in reduction of transmission.

In terms of the potential utility of plasmodial HAP2 as a TBV target/immunogen, the *cd* loop (residues 174–191 in *P. berghei*; 178–195 in *P. falciparum*) is the second section of the protein that has conclusively demonstrated the ability to induce transmission-blocking immunity. Immunization of rabbits with a recombinant fraction of *P. berghei* HAP2 (residues 355–609), containing a region of (predicted) domain II and the majority of domain III, generated polyclonal sera that profoundly inhibited *Plasmodium* fertilization *in vitro* and *ex vivo* (up to 81.1%/34% reduction in intensity/prevalence in the SMFA) (Blagborough and Sinden, 2009). Experiments on the corresponding region of HAP2 in *P. falciparum* using recombinant protein expressed in a wheat germ cell-free expression system (Miura et al., 2013) also resulted in high levels of transmission blockade (97% reduction in intensity). Interestingly, our results suggest that certain regions of the HAP2 ectodomain are incapable of inducing a transmission-blocking response. We examine an “upstream” region of the protein within this study (*P. berghei* residues 123–142) and demonstrate that, although induced serum and antibodies recognize the surface of the gamete by IFA (Figures 1A and 2B), no resulting inhibition in transmission is observed, either following direct immunization (Figure 1B), *in vitro* (Figure 2F), or *ex vivo* (Figures 4A–4C). Considering this, the active mechanisms of functional transmission-blocking antibodies against HAP2 are not currently understood clearly. In the future, further studies to examine immunogenic and functional epitopes of *Plasmodium* HAP2 would be logical and potentially advantageous. The presence of two distinct domains of HAP2 that are capable of mediating transmission-blocking immunity is relevant when considering future design of a potential anti-HAP2 TBV and the potential mechanism of action and immunity of HAP2 in *Plasmodium*. A combination of recombinant protein or viral vectors vaccines containing both the *cd* loop and domain 355–609 (*berghei*) within a single bipartite subunit vaccine could logically result in enhanced efficacy when compared the use of single antigens alone. The immunogens used within this study generated efficacy in *P. falciparum* and *P. berghei* using only a short, simple peptide, conjugated to KLH carrier protein. The small size of the synthetic immunogen used, (although circumventing the challenging nature of heterologous protein expression in *Plasmodium*) (Mehlin et al., 2006) clearly limits the number of functional epitopes that can be generated within a single vaccination. The type of adjuvant, length of peptide, class of carrier protein, and immunization regime was not extensively optimized and still a significant transmission-blocking effect was observed. Further experimentation should examine these variables in order to optimize and enhance the potential efficacy of an anti-HAP2 TBV. This study uses Titermax gold, a readily available pre-clinical adjuvant. It might be argued that studies looking at a wider range of adjuvants (e.g., the clinical saponin-based adjuvants such as Matrix M or Montanide ISA) may give enhanced efficacy. We additionally recognize, especially in the context of well documented cross-virus antibody-mediated neutralization of class II-mediated viral fusion (e.g., between Zika and Dengue) (Rouvinski et al., 2015, Barba-Spaeth et al., 2016, Dejnirattisai et al., 2016), that the observed ability of antibodies raised against the *P. falciparum* HAP2 *cd* loop to initiate a transmission-blocking response in the rodent malaria parasite *P. berghei* raises interesting potential issues regarding the design and synthesis of an anti-malarial TBV with cross-plasmodial species activity in the future. Additional studies could also be expanded to examine the immunogenicity of HAP2 in *P. vivax*, where development of a potent TBV is of growing scientific and clinical interest (Mueller et al., 2015).

The data presented here provides further evidence that HAP2 in *Plasmodium* is a TBV target antigen, but also supports the concept that potent transmission-blocking immunity can be achieved by targeting the male (micro) gamete. Previously, in addition to HAP2, multiple gamete-surface proteins have been shown to induce transmission-blocking efficacy from modest (∼40%) to high (∼100%) levels (P48/45, P47, P230, PSOP12) (Williamson et al., 1995, van Schaijk et al., 2006, Outchkourov et al., 2008, Sala et al., 2015). Due to the comparatively short lifespan, increased fragility, and availability of proven surface-localized antigens on the male gamete of *Plasmodium*, targeting this stage of the parasite life cycle is a promising method of inhibiting parasite transmission. Although P48/45 and P230 are directly implicated in the initial process of male/female gamete adhesion (van Dijk et al., 2001, van Dijk et al., 2010), the successful targeting of HAP2 in multiple studies suggests that targeting the later phases of fertilization, i.e., membrane/gamete fusion, can result in potent antibody-mediated interruption of transmission. To target the sexual stages of the malaria parasite further, a deeper understanding of transmission and specifically, the mechanism of fertilization within *Plasmodium* is advantageous and offers the potential for the development of new, effective vaccines. More broadly, class II fusion proteins are expressed across multiple taxa and species, including in a wide range of organisms of veterinary and clinical importance (e.g., dengue, yellow fever, West Nile viruses, alphaviruses, and Zika) (Podbilewicz et al., 2006, Fédry et al., 2017, Pinello et al., 2017, Valansi et al., 2017). Given the success of the simple strategy outlined here, and the fact that all currently characterized class II fusion proteins utilize similar physical principles and topology to drive membrane fusion, it is not unreasonable to suggest that further studies in these organisms may want to examine the possibility of targeting HAP2, or specifically, the *cd* loop of class II fusion proteins, in order to induce homologous or cross-species therapeutic efficacy.

## Experimental Procedures

### Peptide and Rabbit Anti-HAP2 Antibodies

Peptide regions of HAP2 that correspond to the predicted *cd* fusion loop in *P. falciparum* (*Pf cd* loop, aas 178–195: SYHLFKNDNSIKRAKLKC-KLH) and *P. berghei* (*Pb cd* loop, aas 174–191: TYNYFKDDEFIKRAKLKC-KLH) along with a peptide upstream of the predicted fusion loop in *P. berghei* (*Pb* upstream, aas 123–142: NNHKFEQYTQEQINKYTYTC-KLH) were produced by commercial company Yenzym Antibodies (USA) and C-terminally fused to carrier protein, KLH. Antibodies to these peptides were generated in rabbits, affinity-purified against the relevant peptide used for immunization, and commercially validated by Yenzym by ELISA and western blot.

### General Parasite Maintenance

General parasite maintenance was carried out as described in Ramakrishnan et al. (2013). Briefly, *P. berghei* ANKA 2.34 parasites were maintained in 6- to 8-week-old female Tuck Ordinary (TO) mice (Harlan) by serial mechanical passage (up to a maximum of eight passages). If required, hyper-reticulosis was induced 3 days before infection by treating mice intraperitoneally (i.p.) with 200 μL phenylhydrazinium chloride (PH; 6 mg/mL in PBS; ProLabo UK). Mice were infected i.p. and infections were monitored using Giemsa-stained tail blood smears as described previously (Blagborough et al., 2013b).

### Immunization Regime

Groups of BALB/c (inbred) mice (n = 5) (Harlan, UK) were immunized twice at 3-week intervals with 50 μg and 30 μg of either *Pb*HAP2_123-142_KLH, *Pb*HAP2_174-191_KLH, PfHAP2_178-195_KLH, or KLH-only peptide protein mixed with TiterMax Gold Adjuvant, injected subcutaneously. Sera was collected immediately prior to each injection/boost to evaluate anti-HAP2 response. Immunized mice and non-immunized controls were used to assess transmission-blocking responses by the DFA as described below, following which, sera were collected by terminal anesthesia and cardiac puncture to be used in DFA and SMFA (described below).

### DFA

Routine maintenance of *P. berghei* was carried out as described above. Prior to challenge, mice were PH-treated and 3 days later infected i.p. with 10^6^ *P. berghei* ANKA 2.34. Three days post-infection, animals were anesthetized, and >50 female *Anopheles stephensi* mosquitoes allowed to blood feed on each mouse. Twenty-four hours later, unfed mosquitoes were removed. Mosquitoes were maintained on 8% (w/v) fructose, 0.05% (w/v) p-aminobenzoic acid at 19–22°C, and 50%–80% relative humidity. Day 14 post-feeding, mosquito midguts were dissected and oocyst intensity and prevalence observed by standard phase microscopy and recorded. Reduction in oocyst intensity and prevalence in immunized mice were calculated with respect to KLH-immunized controls.

### *In Vitro* Ookinete Conversion Assay

PH-treated mice were injected with 5 × 10^7^ parasites i.p. On day 3 or 4 of infection, parasitemia was counted on a Giemsa-stained tail blood smear and exflagellation of male gametocytes was checked by addition of a drop of exflagellation medium to a drop of tail blood. Hosts observed to have exflagellating parasites were exsanguinated by cardiac puncture and each 20 μL of blood taken up in 450 μL ookinete medium. Individual cultures were then added to pre-prepared 24-well plates (Nunc) containing anti-HAP2 rabbit sera or anti-UPC10 (negative control), and incubated for 24 hr at 19°C. Cultures were harvested after 24 h rby centrifugation (500 × *g*, 5 min), washed once in 100 μL ookinete medium, and the pellet taken up in 50 μL ookinete medium containing Cy3-conjugated Pbs28 monoclonal antibody (mAb) clone 13.1 (1:500). Ookinetes and macrogametocytes were then immediately counted by fluorescence microscopy. Ookinete conversion rates were calculated as described previously (Blagborough et al., 2013b). Results were collated from three separate experiments and inhibition expressed as the percentage reduction in ookinete conversion with respect to the anti-UPC10 control.

### SMFA

Female *An. stephensi* (SDA 500 strain) were starved for 24 hr and then fed on heparinized *P. berghei*-infected blood using SMFAs (Blagborough and Sinden, 2009). For each feed, 350 μL of *P. berghei* ANKA 2.34-infected blood containing asexual and sexual stages of the parasite was mixed with 150 μL of PBS containing either anti-*Pb cd* loop, anti-*Pb* upstream, or the isotopic mAb UPC10 (negative control) (Sigma) antibodies to yield final antibody concentrations of 250 and 500 μg/mL. Data demonstrating the inability of both UPC10 and rabbit affinity purified antibodies to induce transmission-blocking activity is presented in Table S1. Mosquitoes were handled, maintained, and analyzed as described above. Reductions in oocyst intensity and prevalence was calculated with respect to control feeds as described in Blagborough and Sinden (2009).

### DMFA

To determine transmission-blocking efficacy against field isolates of *P. falciparum*, children between the ages of 5–11 years in Bobo-Dioulasso, Burkina Faso, were screened for the presence of *P. falciparum* gametocytes by thick blood smears. Gametocytemias of 32, 72, and 112 per μL patient blood were examined to study efficacy at a wide range of parasitic densities. 10 mL blood was drawn in heparinized tubes to obtain gametocytes. The plasma from the gametocyte-positive donor blood was replaced by AB+serum from a European donor, the test (or control) serum was added at the stated dilution in the final mixture, and immediately fed to pots of *An. colluzi* mosquitoes via a parafilm membrane at 37°C. DMFA experiments were performed using non-heat-inactivated AB+ serum. The full fed females were sorted and maintained in cages at 28°C ± 2, 80% ± 05 relative humidity (RH), with 10% glucose solution. Mosquitoes were dissected day 7 post-feeding in a drop of 0.5% mercurochrome with midguts examined for oocysts by light microscopy. Intensity, prevalence, and reduction in both was calculated as described in Blagborough and Sinden (2009).

### IFA

Exflagellation of male gametocytes was monitored by adding 10 μL tail blood from a high-gametocytemia mouse into 10 μL ookinete medium (RPMI1640 containing 25 mM HEPES, 20% fetal calf serum (FCS), 100 μM xanthurenic acid [pH 7.4]) and observing exflagellation centers under a light microscope with a 40× objective as described previously (Sebastian et al., 2012). Exflagellating gametocytes were fixed in solution and prepared for IFA using 4% paraformaldehyde in PBS. Primary antisera in 3% BSA/PBS included mouse serum from DFA (1:300), rabbit antibodies (1:300), and mouse anti-GFP (Thermo Fisher Scientific) (1:1,000). Following washes, appropriate secondary antibodies (Alex Fluor-488, Molecular Probes) were used at 1:500 before mounting in VectaShield with DAPI (Vector Laboratories). Fluorescence images were obtained using a fluorescence microscope 60× objective on an EVOSFL Cell Imaging System (Life Technologies). Image handling was undertaken using either ImageJ or Adobe Photoshop CC. Final images were assembled in Adobe Illustrator CC for figure generation.

### Statistical Analysis

Statistical analysis was performed on the entire cohort of each experimental group using GraphPad Prism. For DFA, SMFA, and DMFA, significance was assessed using Mann-Whitney U (to examine differences in intensity) and Fisher’s exact probability tests (to examine differences in prevalence). Parametric ELISA tests were assessed using t test. p values < 0.05 were considered statistically significant (^∗∗∗^p ≤ 0.0001, ^∗∗∗^p = 0.001, ^∗∗^p = 0.001–0.01, ^∗^p = 0.01–0.05).

### Ethical Statement

All procedures were performed in accordance with the UK Animals (Scientific Procedures) Act (PPL 70/8788) and approved by the Imperial College AWERB. The Office of Laboratory Animal Welfare Assurance for Imperial College covers all Public Health Service supported activities involving live vertebrates in the United States (no. A5634-01). For field studies, informed consent from parents or guardians was obtained for children positive for gametocytes prior to blood sampling/DMFA (Protocol 003-2009/CE-CM, Centre Muraz Institutional Ethical Committee).

## Author Contributions

F.A., W.J.S., and A.M.B. conceived the work. A.M.B. and W.J.S. acquired funding. Investigation performed by F.A., A.M.B., K.S., and D.F.D. Data analysis by F.A., A.M.B., and W.J.S. Writing – original draft by F.A., W.J.S., and A.M.B. Writing – review and editing by K.A.S., D.F.D., J.L., J.P., and N.V.G.

## Supplemental Information

Table S1. SMFA Comparison of UPC10 and Rabbit IgG The mean (from three replicates) changes in intensity (mean number of oocysts per midgut) and prevalence with concentrations of UPC10 and rabbit IgG at 500 μg/ml were calculated with respect to PBS control and each other. P < 0.05, Mann-Whitney *U* test P < 0.05, Fisher’s exact test.
